# Characterisation of the antibacterial properties of the recombinant phage endolysins AP50-31 and LysB4 as potent bactericidal agents against *Bacillus anthracis*

**DOI:** 10.1038/s41598-017-18535-z

**Published:** 2018-01-08

**Authors:** Sangjin Park, Soo Youn Jun, Chang-Hwan Kim, Gi Mo Jung, Jee Soo Son, Seong Tae Jeong, Seong Jun Yoon, Sang Yup Lee, Sang Hyeon Kang

**Affiliations:** 1 Metabolic and Biomolecular Engineering National Research Laboratory, Department of Chemical and Biomolecular Engineering (BK21 Plus Program), Center for Systems and Synthetic Biotechnology, Institute for the BioCentury, Korea Advanced Institute of Science and Technology (KAIST), Daejeon, 34141 Republic of Korea; 2 The 5th R&D institute, Agency for Defense Development (ADD), Yuseong P.O.Box 35-5, Daejeon, 34186 Republic of Korea; 3 iNtRON Biotechnology, Inc., Room 903, JungAng Induspia V, 137, Sagimakgol-ro, Jungwon-gu, Seongnam-si, Gyeonggi-do, 13202 Republic of Korea

## Abstract

The recombinant phage endolysins AP50-31 and LysB4 were developed using genetic information from bacteriophages AP50 and B4 and were produced by microbial cultivation followed by chromatographic purification. Subsequently, appropriate formulations were developed that provided an acceptable stability of the recombinant endolysins. The bacteriolytic properties of the formulated endolysins AP50-31 and LysB4 against several bacterial strains belonging to the *Bacillus* genus including *Bacillus anthracis* (anthrax) strains were examined. AP50-31 and LysB4 displayed rapid bacteriolytic activity and broad bacteriolytic spectra within the *Bacillus* genus, including bacteriolytic activity against all the *B*. *anthracis* strains tested. When administered intranasally, LysB4 completely protected A/J mice from lethality after infection with the spores of *B*. *anthracis* Sterne. When examined at 3 days post-infection, bacterial counts in the major organs (lung, liver, kidney, and spleen) were significantly lower compared with those of the control group that was not treated with endolysin. In addition, histopathological examinations revealed a marked improvement of pathological features in the LysB4-treated group. The results of this study support the idea that phage endolysins are promising candidates for developing therapeutics against anthrax infection.

## Introduction

*Bacillus anthracis* (anthrax) is a bacterium of significant concern owing to its potential uses in bioterrorism and biowarfare. Currently, the medical countermeasure recommended for anthrax infection is the administration of antibiotics. Some strains of *B*. *anthracis*, however, show natural antibiotic resistance^1,2^. In addition, *in vitro* development of antibiotic resistance was demonstrated in a laboratory^3^. Strains that were even resistant to ciprofloxacin were generated by *in vitro* selection^4,5^. In the late 1980s, scientists of the former Soviet Union allegedly made a strain resistant to five antibiotics^6^. Therefore, there have been concerns about bioterrorism through the use of engineered antibiotic-resistant *B*. *anthracis* strains^7–9^. Despite such a potentially significant public health threat, there are currently no antibacterial therapeutic measures, while treatment with anti-toxin antibodies is a potential alternative^10^. Therefore, it is necessary to secure novel antibacterial agents and biodefence tools to respond to a potential future *B*. *anthracis* crisis. These novel antibacterial agents should be effective against both antibiotic-susceptible and antibiotic-resistant *B*. *anthracis* strains. To meet this requirement, the novel antibacterial agents should have a completely different mode of action than those of commonly used antibiotics. Additionally, because anthrax is an acute infectious disease, rapid bacteriolytic activity is required. Based on these requirements, phage endolysins have been proposed as candidate novel antibacterial agents for the specific use against *B*. *anthracis* ^11,12^.

Phage endolysins (also known as phage lysins or lysins) are enzymes of bacteriophages that degrade the peptidoglycan wall of infected bacteria, resulting in the release of progeny phages^13^. The exogenous application of the purified recombinant phage endolysin to Gram-positive bacteria induces rapid bacterial cell lysis and death^13,14^. Phage endolysins are promising antibacterial agents to use against pathogens because of their high specificity and low level of bacterial resistance development^15^. They are also promising antibacterial agents to use against antibiotic-resistant pathogens. Several prior studies have demonstrated the efficacy of endolysins against *B*. *anthracis* ^11,12^, *Streptococcus pneumonia* ^16^, *Staphylococcus aureus* ^17^, and *Clostridium difficile* ^18^, and animal model studies have indicated their potential to cure human infections^16,19–25^.

Phage endolysins differ from standard antibiotics in their potency, specificity, speed, and activity against antibiotic-resistant bacteria. Additionally, phage endolysins are usually specific for a particular target bacterial species and do not generally lyse non-target bacteria, including commensal bacteria, that may reduce clinical complications^26^. Thus, research on phage endolysins is one of the most promising areas on the development of novel antibacterials against human pathogens^26^. In this paper, we report on the development of novel antibacterial agents effective in treating *B*. *anthracis* infections through bacteriolytic activity based on phage endolysins.

## Results

### Selection of target endolysins

Based on publicly available genetic information for endolysins, we chose two phage endolysins: those of *B. anthracis* bacteriophage AP50^27^ and *B*. *cereus* bacteriophage B4^28,29^. Phage endolysin AP50-31 was developed using genomic information from *B*. *anthracis* bacteriophage AP50, and phage endolysin LysB4 was developed using genomic information from *B*. *cereus* bacteriophage B4. Bacteriophage AP50 has two putative endolysin genes (ORFs 25 and 31) and the ORF 31 was selected due to its unique domain type. AP50-31 is predicted to have the amidase02_C and amidase_3 (N-acetylmuramoyl-l-alanine amidase) catalytic domains while lacking a cell wall binding domain. LysB4 is predicted to have a catalytic VanY (l-alanoyl-d-glutamate endopeptidase) domain and a cell wall binding SH3_5 domain^28^. AP50-31 and LysB4 do not have any signal peptides or transmembrane regions based on the analyses using SignalP^30^ and THMHH^31^ prediction programs. The characteristics of AP50-31 and LysB4 are presented in Supplementary Table S1.

To understand the relationships between the target endolysins in this study and other phage endolysins, 23 putative endolysins were selected from *Bacillus* phages that were assigned as completely sequenced in the NCBI database. A phylogenetic tree was constructed for the 25 endolysins, including AP50-31 and LysB4 (Fig. S1). The phylogenetic tree placed AP50-31 into a group between PBC1 and BCJA1c, while LysB4 was determined to belong to SPO1, forming a cluster with Spock, AvesoBmore, Riley, and Troll. The amino acid sequences of endolysins LysB4^29^, Spock^32^, Troll^33^, AvesoBmore^34^, and Riley^33^ were almost identical, although their parent bacteriophages were different (Fig. S2). The characteristics of the putative endolysins used in the construction of phylogenetic tree are presented in Supplementary Table S1.

### Production and purification of AP50-31 and LysB4

Expression plasmids were constructed based on the pBAD expression system and were transformed with *E*. *coli* BL21 to produce the recombinant endolysins AP50-31 and LysB4 (Table 1). Recombinant phage endolysins were produced using these production hosts.

**Table 1:** Bacterial strains and plasmids used in this study.

Strain or plasmid	Relevant characteristics	Reference or source
**Strains**		
***E***. ***coli***		
BL21	Host for recombinant endolysin expression	Novagen (Madison, WI, USA)
**Non-** ***anthracis Bacillus***		
*Bacillus cereus* ATCC 4342	Isolate from milk	ATCC
*Bacillus circulans* ATCC 21783	Isolate from soil	ATCC
*Bacillus laevolacticus* ATCC 23492	Isolate from *Ranunculus sceleratus*	ATCC
*Bacillus licheniformis* KCOM 1491	Isolate from human subgingival dental plaque	KCOM
*Bacillus megaterium* ATCC 10778	Isolate from soil	ATCC
*Bacillus pumilus* KCTC 3713	Isolate from air-conditioner filter of subway station	KCTC
*Bacillus subtilis* RIK 1285	*Bacillus* expression host strain	Takara (Kusatsu, Japan)
*Bacillus thuringiensis* BGSC 4AA1	Wild type isolate	BGSC
*Bacillus thuringiensis* BGSC 4AJ1	Wild type isolate	BGSC
*Bacillus thuringiensis* BGSC 4BA1	Wild type isolate	BGSC
*Bacillus thuringiensis* BGSC 4CC1	Wild type isolate	BGSC
***B***. ***anthracis***		
ΔSterne	pXO1^−^ pXO2^−^	Hanyang University (Seoul, Republic of Korea)
Sterne	pXO1^+^ pXO2^−^	KCDC
ATCC 14578	pXO1^+^ pXO2^+^	KCDC
HYU01	pXO1^+^ pXO2^+^	Hanyang University^49^
**Plasmids**		
pBAD::SAL-1	Backbone vector used for the construction of pBAD-AP50-31 and pBAD-B4	^46^
pBAD-AP50-31	Endolysin AP50-31 expression plasmid	This study
pBAD-B4	Endolysin LysB4 expression plasmid	This study

ATCC, American Type Culture Collection (Manassas, VA, USA); KCOM, Korean Collection for Oral Microbiology (Gwangju, Republic of Korea); KCTC, Korean Collection for Type Cultures (Jeongeup, Republic of Korea); BGSC, Bacillus Genetic Stock Center (Columbus, OH, USA); KCDC, Korea Centers for Disease Control and Prevention (Osong, Republic of Korea).

AP50-31, with no extraneous amino acid residues such as purification tags, was produced in a soluble form by decreasing the culture temperature to 30 °C immediately after the induction of expression. Using one-step cation-exchange chromatography, AP50-31 was purified from crude *E*. *coli* cell extracts with more than 90% purity, as confirmed by sodium dodecyl sulfate polyacrylamide gel electrophoresis (SDS-PAGE) and size-exclusion chromatography/high-performance liquid chromatography (SEC-HPLC) analyses. This purity was sufficient for all of the experiments performed in this study. The appropriate pH range for binding to the column was narrow under the chromatographic conditions utilised in this study and was within less than ±0.5 deviation from pH 7.5. Using the expression and purification methods described in the Materials and Methods, approximately 72 mg of purified AP50-31 was routinely obtained from 1L of culture.

A soluble form of recombinant LysB4 was successfully produced without decreasing the culture temperature from 37 °C. The endolysin contained no extraneous amino acids. The expression level of LysB4 was similar to that of AP50-31. LysB4 was obtained using a one-step cation-exchange chromatography procedure with more than 90% purity, as confirmed by SDS-PAGE and SEC-HPLC analyses. A total of 132 mg of phage endolysin LysB4 was generally obtained from 1 L of culture.

### Development of formulations adequate for the stable storage of AP50-31 and LysB4

We aimed to develop effective formulations that prevented the aggregation of the phage endolysins while still maintaining stability during 12 weeks of storage and also during a shorter period of physical stress such as agitation. An appropriate formulation for the stable storage and handling of LysB4 was developed through the preformulation screening of pH, buffer type, surfactant and additive while considering the commonly used drug compositions used for injection. The best formulation was comprised of 0.1% (w/v) polysorbate 80, 5% (w/v) sorbitol, and 10 mM l-histidine (pH 6). This formulation significantly stabilised LysB4 that showed nearly 100% integrity and biological activity after 12 weeks of storage at 4 °C and −20 °C or after constant vigorous agitation for up to 4 h. However, this formulation did not provide acceptable stability for storage at 25 °C, because significant degradation was observed after three weeks of storage. Although all of the formulation candidates tested did not provide sufficient stability for AP50-31 when stored at 4 °C, the formulation containing 0.5% (w/v) poloxamer 188, 5% (w/v) sorbitol, and 10 mM sodium phosphate (pH 7.5) provided relatively good stability. This formulation provided sufficient stability for AP50-31 after incubation under accelerated stability study conditions (40 °C for 16 h) under vigorous constant agitation (2,500 rpm) for up to 4 h, and after being stored at −20 °C for 12 weeks. Because AP50-31 was shown to be stable in the three conditions described above, we believed that the formulation containing 0.5% (w/v) poloxamer 188, 5% (w/v) sorbitol, and 10 mM sodium phosphate (pH 7.5) was sufficient to conduct the experiments planned in this study. Thus, the formulation containing 0.5% (w/v) poloxamer 188, 5% (w/v) sorbitol, and 10 mM sodium phosphate (pH 7.5) was chosen as a temporary formulation for the subsequent experiments in this study.

### *In vitro* bacteriolytic properties

The bacteriolytic activities of AP50-31 and LysB4 were examined using a conventional turbidity reduction assay that measured the time required to reach one-half of the starting absorbance (TOD_50_) after each phage endolysin was added at a final concentration of 5 μg/mL to a bacterial cell suspension (optical density at 600 nm [OD_600_] = 1.0). The results obtained for the non-*anthracis Bacillus* strains are summarised in Table 2. AP50-31 and LysB4 displayed broad bacteriolytic spectra within the *Bacillus* genus and displayed rapid bacteriolytic properties. However, differences in bacteriolytic activity and the bacteriolytic spectrum range between the two endolysins were observed. LysB4 exhibited more rapid bacteriolytic activity, and a broader bacteriolytic spectrum range than the phage endolysin AP50-31. Additionally, AP50-31 and LysB4 exhibited rapid and effective bacteriolytic activities against all of the strains of *B*. *anthracis* tested (Table 3). Although LysB4 showed smaller TOD_50_ values against all four *B*. *anthracis* strains tested, the difference was statistically significant only for *B*. *anthracis* ATCC 14578 and *B*. *anthracis* HYU01 (Welch’s t-test, *P* < 0.05).

**Table 2:** Susceptibility of *Bacillus* strains to phage endolysins AP50-31 and LysB4.

Species	Strain	TOD_50_ (min)	
		AP50-31	LysB4
*Bacillus cereus*	ATCC 4342	3.8 ± 0.06	2.2 ± 0.06
*Bacillus circulans*	ATCC 21783	ND	3.5 ± 0.15
*Bacillus laevolacticus*	ATCC 23492	ND	1.4 ± 0.06
*Bacillus licheniformis*	KCOM 1491	19.4 ± 0.21	2.1 ± 0.06
*Bacillus megaterium*	ATCC 10778	NS	2.2 ± 0.06
*Bacillus pumilus*	KCTC 3713	ND	2.6 ± 0.06
*Bacillus subtilis*	RIK 1285	15.0 ± 0.06	0.5 ± 0.06
*Bacillus thuringiensis*	BGSC 4AA1	NS	4.2 ± 0.06
	BGSC 4AJ1	2.2 ± 0.06	NS
	BGSC 4BA1	2.8 ± 0.06	NS
	BGSC 4CC1	2.2 ± 0.00	NS

ND (not determined): TOD_50_ could not be determined under this experimental condition, but susceptibility to the corresponding endolysin was confirmed under a concentration of more than 5 μg/mL; NS (not susceptible): TOD_50_ could not be determined up to 300 μg/mL of phage endolysin. Data are the mean ± standard deviation. The tests were performed three times independently.

**Table 3:** Susceptibility of *B*. *anthracis* strains to phage endolysins AP50-31 and LysB4.

Strain	Virulence plasmid	TOD_50_ (min)	
		AP50-31	LysB4
*B*. *anthracis* ΔSterne	None	9.0 ± 1.73	6.3 ± 0.29
*B*. *anthracis* Sterne	pXO1	9.3 ± 0.76	8.2 ± 1.04
*B*. *anthracis* ATCC 14578	pXO1, pXO2	16.3 ± 2.02	10.2 ± 0.58
*B*. *anthracis* HYU01	pXO1, pXO2	9.5 ± 1.00	7.7 ± 0.29

Data are the mean ± standard deviation. The tests were performed three times independently.

### *In vivo* efficacy test of LysB4 against *B*. *anthracis*

**Figure 1:**
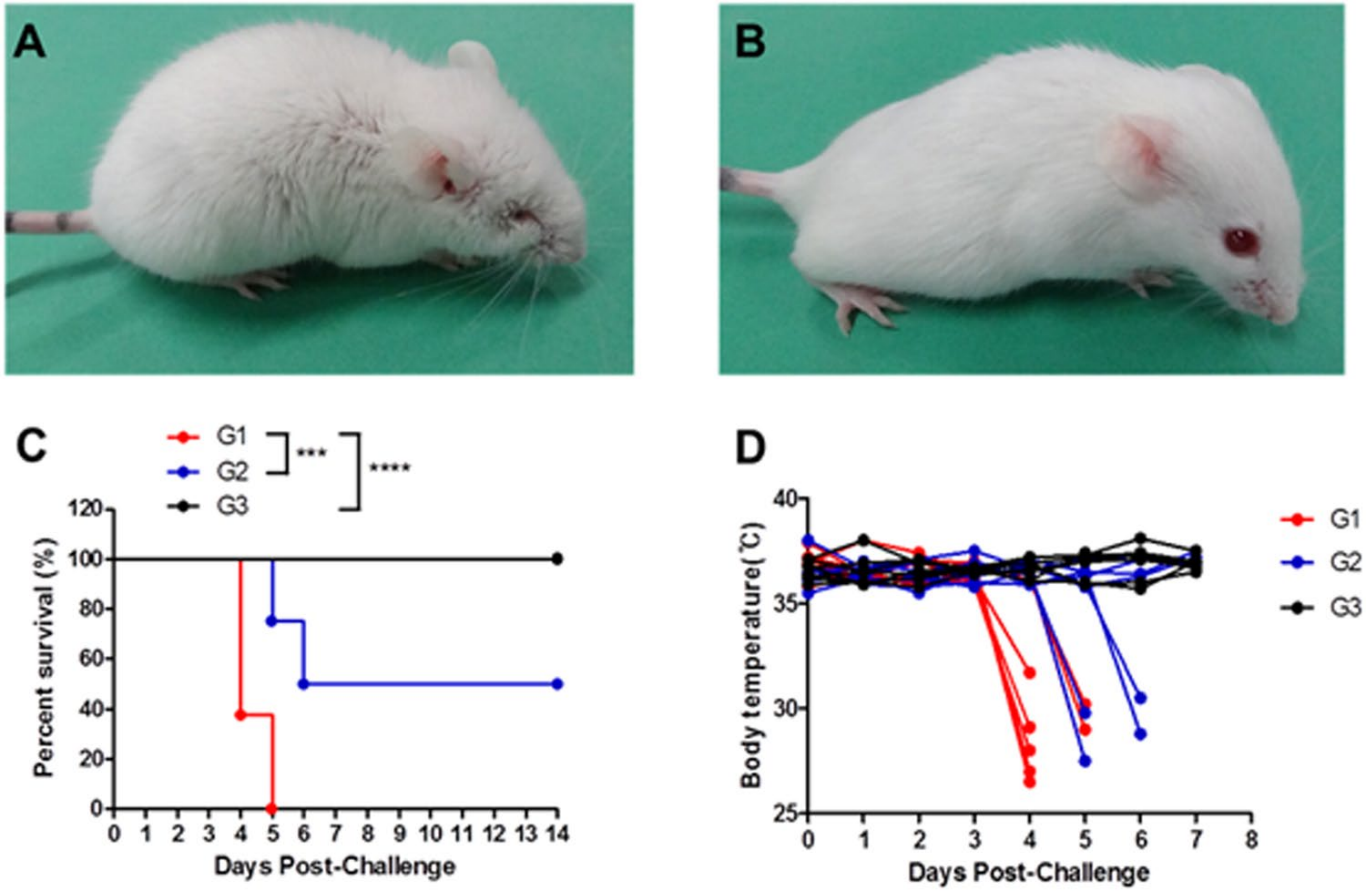
Results of survival rate and body temperature in *in vivo* study. A/J mice were infected intranasally with approximately 4 times the 50% lethal dose of spores of *B*. *anthracis* Sterne. At 6, 24, and 48 h post-infection, buffer (G1) or endolysin LysB4 (G2: 10 μg/head, G3: 100 μg/head) were administered intranasally (8 mice per group). Clinical signs of buffer- (**A**) or LysB4- (**B**) treated mice, survival rate (**C**), and body temperature (**D**) were observed. ***P < 0.001 compared to the G1 group.

Challenge with spores of *B*. *anthracis* caused physical signs of ill health in the buffer-treated mice that manifested as hunched backs, ruffed fur, piloerection, and ocular discharge (G1, Fig. 1A). These clinical signs appeared 3–4 days after the bacterial challenge and became aggravated in a time-dependent manner. LysB4 treatment markedly improved the clinical signs and no symptoms were observed in the high-dose LysB4-treated group (G3, 100 μg/head; Fig. 1B). In addition, LysB4 treatment efficiently rescued the infected mice, and no deaths were observed in the high-dose LysB4-treated group throughout the experimental period. The administration of high-dose LysB4 at 6, 24, and 48 h post-infection provided 100% survival (G3, 100 μg/head), while low-dose LysB4 treatment (G2, 10 μg/head) delayed the onset of death and significantly improved the survival rate (Fig. 1C). The mean body temperature of the mice decreased corresponding to the onset of clinical signs, and the decrease was more prominent in the buffer-treated group compared with the LysB4-treated groups (Fig. 1D).

**Figure 2:**
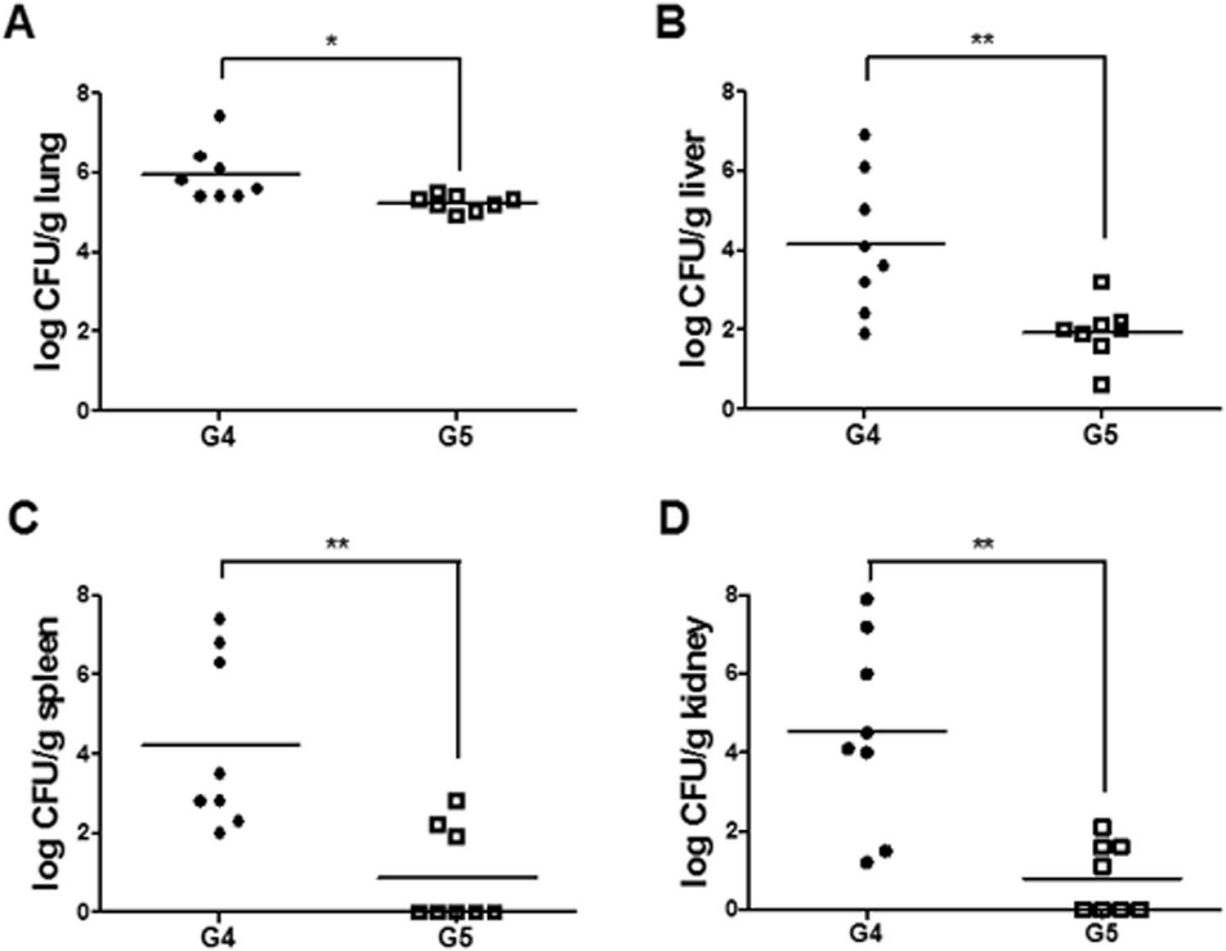
Results of bactrial killing activity of LysB4 in *in vivo* study. *B*. *anthracis*-infected mice were treated with buffer (G4) or LysB4 (G5: 100 μg/head) at 6, 24, and 48 h post-infection and euthanized at day 3 post-infection (8 mice per group). Serial dilutions of lung, liver, spleen, and kidney homogenates were plated on tryptic soy agar plates and incubated at 37 °C overnight, and the colonies were counted. *P < 0.05, **P < 0.01 compared to G4 group.

**Figure 3:**
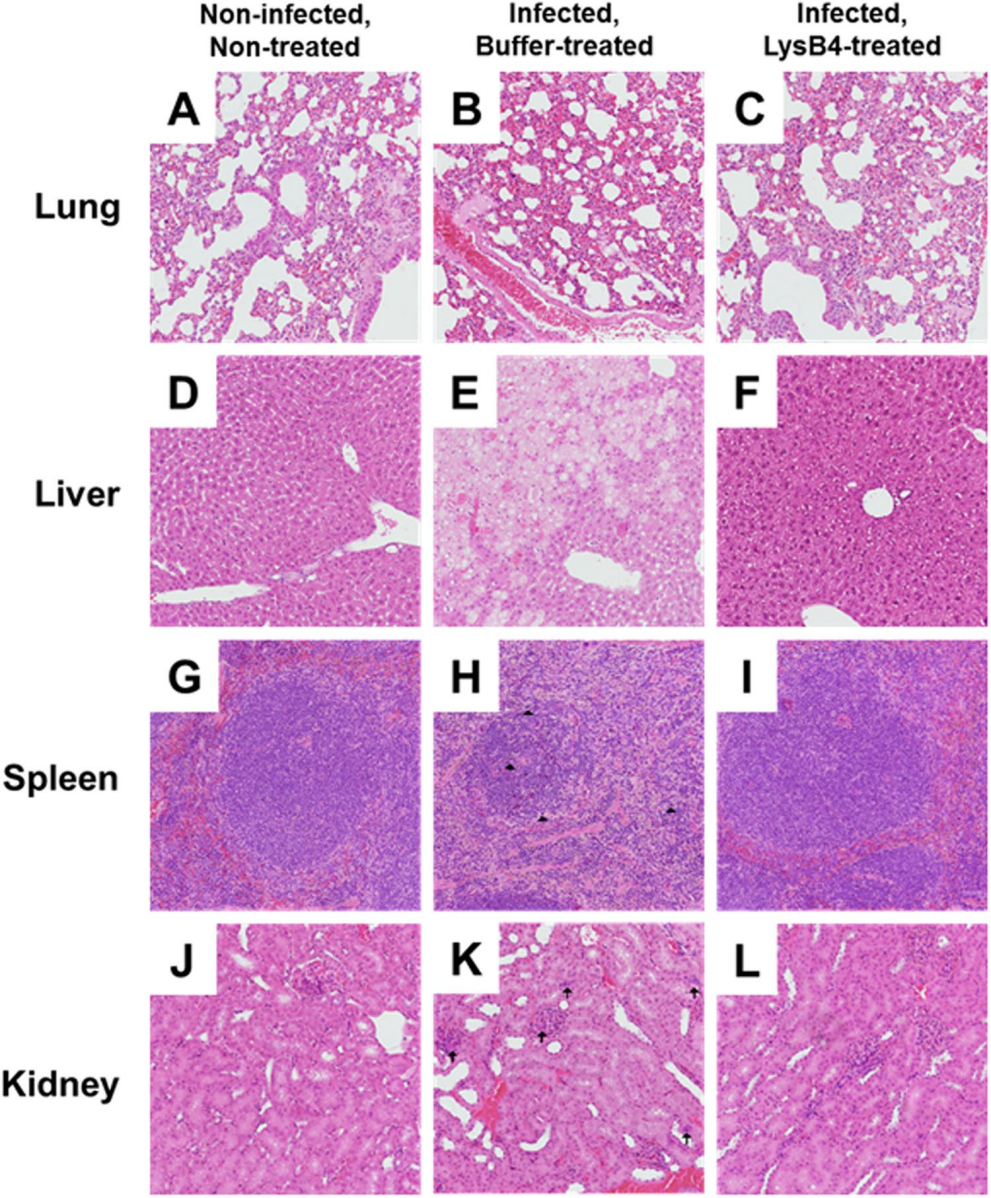
Results of histopathological analysis in *in vivo* study. Organs of the non-infected and non-treated control, the infected and buffer-treated, and the infected and LysB4-treated mice were observed under light microscopy and representative histologic images of the lungs (**A**–**C**), livers (**D**–**F**), spleens (**G**–**I**), and kidneys (**J**–**L**) are shown. Arrowheads indicate apoptotic lymphocytes and arrows indicate clusters of bacilli. Haematoxylin and eosin staining; magnification × 200.

To determine the effect of LysB4 on bacterial clearance, the lungs, livers, spleens, and kidneys were extracted from buffer- or LysB4-treated mice at day 3 post-infection and the total count of bacteria (spores and vegetative cells) in each tissue was determined. In all of the tissues analysed, the bacterial count of the LysB4-treated group was significantly lower than that of the buffer-treated group. In the LysB4-treated group, most of the bacteria were cleared in the liver, spleen, and kidney, but a large number of the bacteria remained in the lung (Fig. 2). Histological observations revealed no remarkable pathological findings in the organs of the non-infected and non-treated control mice (Fig. 3A,D,G and J). In the lung tissue of the infected and buffer-treated group, the alveolar septum was slightly expanded and some alveolar spaces were filled with proteinaceous materials (Fig. 3B). In addition, there was marked vacuolar degeneration and necrosis of hepatocytes in the liver (Fig. 3E). Apoptotic lymphocytes characterised by fragmented and condensed nuclear debris were visible throughout the spleen in the infected and buffer-treated group (Fig. 3H). In addition, lymphocytes were significantly depleted in the white and red pulp of the spleen, and the sheath of the marginal zone was reduced (Fig. 3H). In the kidney of the infected and buffer-treated group, myriad bacilli were frequently observed in the glomeruli, tubules, and interstitial tissues (Fig. 3K). However, most of these pathological findings were markedly improved in the organs of the infected and LysB4-treated group (Fig. 3C,F,I and L), indicating that LysB4 had a protective effect against *B*. *anthracis* infection in mice.

## Discussion

With the rapid emergence of antibiotic-resistant bacteria worldwide, there is a need for new and innovative antibacterial agents with different modes of action than those of commonly used antibiotics. For *B*. *anthracis*, a potential biological weapon of mass destruction^7,35^, fears related to the possible engineering of antibiotic resistance necessitate a need to develop new bacteriolytic antibacterial agents. Phage endolysins are a potentially good candidate to act as antibacterial agents against *B*. *anthracis* ^11^.

Although many recombinant phage endolysins show a poor expression or insolubility when produced in their native forms^36^, the phage endolysins AP50-31 and LysB4 used in this study were successfully produced in their soluble forms and purified with high yield and purity. Compared to the previously reported production of endolysin LysB4 containing an N-terminal His-tag for purification^28^, the endolysins used in this study do not contain any extra amino acid residues including the His-tag, thus providing additional advantages for their use as therapeutic agents for use in human applications. The recombinant phage endolysins AP50-31 and LysB4 displayed broad bacteriolytic spectra within the *Bacillus* genus with different host susceptibilities and bacteriolytic activities. More importantly, they exhibited rapid bacteriolytic activities against all of the strains of *B*. *anthracis* tested, demonstrating their potential as antibacterial agents for *B*. *anthracis* infections. AP50-31 and LysB4 showed different TOD_50_ values against the same bacterial strain in the *in vitro* bacteriolytic activity experiment.

AP50-31 has 252 amino acids, and its molecular mass is 27.7 kDa. The theoretical isoelectric point of AP50-31 is estimated to be 6.44, and it contains catalytic amidase02_C and amidase_3 (N-acetylmuramoyl-l-alanine amidase) domains. In comparison, LysB4 has 262 amino acids, and its molecular mass is 27.9 kDa. The theoretical isoelectric point of LysB4 is estimated to be 9.21, and it has a catalytic VanY domain and a cell wall binding SH3_5 domain^28^. Although AP50-31 and LysB4 originate from bacteriophages and have similar molecular masses, there are significant differences between their isoelectric points and types of catalytic domains. We assume that these differences are the reason for the differences in TOD_50_ values observed for the two endolysins evaluated in this study. Additional research should be performed to analyse the structure-function relationships in detail.

This research represents the first report of the results of *in vivo* endolysin efficacy tests against *B*. *anthracis* using an animal model of *B*. *anthracis* infection. Previous *in vivo* studies used *B*. *cereus* ATCC 4342 (also known as RSVF1) as a surrogate for *B*. *anthracis*. *B*. *cereus* ATCC 4342 was isolated from milk and is categorised as a biosafety level 1 microorganism. This strain is frequently used as a surrogate for *B*. *anthracis* in biodefence studies. It was used as a bacterial challenge agent for *in vivo* experiments on endolysin PlyG^11^ and endolysin PlyPH^12^. Although the results of the *in vivo* experiments with *B*. *cereus* ATCC 4342 are valuable, the results obtained using this surrogate need careful interpretation since the pathogenic mechanisms of *B*. *cereus* and *B*. *anthracis* are substantially different. In particular, *B*. *cereus* ATCC 4342 lacks the pXO1 virulence plasmid that encodes unique anthrax toxins that include the protective antigen, lethal factor, and edema factor. For this reason, *B*. *anthracis* needs to be used for animal infection studies to prove the therapeutic effect of endolysins against anthrax. In addition, although growing vegetative cells of *B*. *cereus* were used for infection in previous studies, to simulate the actual situation, the use of spores is more suitable, since spores are expected to be the primary means of attack in potential bio-terrorism incidents involving anthrax^37^. Among the many routes of infection, the intranasal route was chosen since inhalational anthrax is the form of anthrax that would be most likely to be used in a bioterrorism attack to produce high mortality^7^. The intranasal route was chosen to administer the endolysin because we reasoned that delivering a therapeutic agent by the same route that infectious agents exploits to enter its host would significantly increase the probability of contact between the therapeutic and infectious agents. Indeed, the intranasal route of endolysin administration was effective, since delivering 100 μg of LysB4 at 6, 24, and 48 h post-infection saved 100% of the infected mice from death (Fig. 1C).

Compared to other tissues, a large number of bacteria remained in the lung (Fig. 2). Because the number of bacterial counts in the lung was the highest in the buffer-treated group (G4), and the spores were introduced intra-nasally, the higher number of bacterial counts observed in the lung in the endolysin-treated group (G5) might not be abnormal. However, an adequate explanation of this phenomenon was not possible until now. High variability in the number of remaining bacteria between individual mice was observed especially in the livers, spleens, and kidneys of the control G4 group (Fig. 2). This variability was also reported in other murine inhalational anthrax studies^38–40^.

We wish to emphasise that this is the first study to demonstrate the *in vivo* efficacy of endolysin against anthrax. However, our research lacks three experimental points that will be needed to fully evaluate the therapeutic potential of endolysin for treating human anthrax infection. First, the strain used for infection was *B*. *anthracis* Sterne. This strain, chosen for safety reasons, is an animal vaccine strain lacking the capsule-forming pXO2 plasmid that is necessary for full virulence. Even though the use of *B*. *anthracis* Sterne with A/J mice is a well-established model of anthrax infection, a fully virulent strain (pXO1^+^/pXO2^+^) is more desirable to show therapeutic efficacy. Second, a mouse model alone cannot fully simulate a human infection in many respects^41^. Various animal models are used in anthrax research, and each has its own advantages and disadvantages as a model of human anthrax^41^. Thus, the efficacy of endolysin needs to be tested in other animal models (ideally in a non-human primate model). Third, an aerosol challenge model is preferable to an intranasal challenge model to study the pathogenesis of inhalational anthrax^42^. To develop endolysins as therapeutics for human use, future studies should address these three aspects that could not be addressed in this study due to safety issues and a lack of highly specialised equipment and facilities.

Nonetheless, in this study we showed that the intranasal administration of the phage endolysin LysB4 could protect mice from lethal anthrax infections. We expect that the results and insights provided in this paper will assist in the development of phage endolysin-based therapeutics for use against anthrax in the future.

## Materials and Methods

### Selection of target phage endolysin sequences

The aim of this study was to develop phage endolysins that have antibacterial activity against *B*. *anthracis*. From the NCBI website (http://www.ncbi.nlm.nih.gov/genome/), we collected the genetic information of bacteriophages specific for *B*. *anthracis*, *B*. *cereus*, and *B*. *thuringiensis*. We included *B*. *cereus* and *B*. *thuringiensis* because they are close relatives of *B*. *anthracis* ^43^ and are often used as surrogates for *B*. *anthracis* ^44,45^. The genetic information from prophages was excluded. From the collected genetic information, the putative endolysin sequences were extracted. Subsequently, the target sequences of putative endolysins were selected based on an analysis of the types of catalytic domain of the putative phage endolysins using the NCBI BLASTP program (http://blast.ncbi.nlm.nih.gov/Blast.cgi).

### Construction of expression hosts for the recombinant phage endolysins AP50-31 and LysB4

We chose the putative endolysin sequences of bacteriophage AP50 and bacteriophage B4 because bacteriophage AP50 is a *B*. *anthracis* bacteriophage^27^, and bacteriophage B4 is a *B*. *cereus* bacteriophage^28,29^ based on several criteria. The putative endolysin sequences of bacteriophage AP50 and bacteriophage B4 have low similarity with other endolysin sequences reported to have the anti-*B*. *anthracis* activity. In addition, the anti-*B*. *anthracis* activity of the phage endolysins of bacteriophage AP50 and bacteriophage B4 has not yet been reported. Using the genetic information available for bacteriophage AP50 (GenBank accession number EU408779) and bacteriophage B4 (GenBank accession number JN790865), the expression plasmids pBAD-AP50-31 and pBAD-B4 were constructed by inserting the genes encoding the endolysins of bacteriophage AP50 and bacteriophage B4, respectively, into pBAD::SAL-1^46^. The nucleotide sequences used to construct pBAD-AP50-31 and pBAD-B4 are the sequences with the GenBank gene ID numbers 7018716 and 13828781, respectively. Oligonucleotides containing the genes encoding the endolysins of bacteriophages AP50 and B4 were prepared by chemical synthesis and inserted into the modified pBAD::SAL-1 plasmid by pre-incorporating an *Nco*I site at the translational start codon of pBAD::SAL-1 using the *Nco*I and *Not*I sites. The resulting plasmids were designated pBAD-AP50-31 and pBAD-B4, respectively, and were used as expression plasmids for the phage endolysins. *E*. *coli* BL21 cells were transformed with pBAD-AP50-31 and pBAD-B4, and the transformants were designated *E*. *coli* BL21-pBAD-AP50-31 and *E*. *coli* BL21-pBAD-B4, respectively. These cells were used as production hosts for the recombinant phage endolysins. The target phage endolysins were designated phage endolysin AP50-31 and phage endolysin LysB4.

### Phylogenetic and bioinformatic analyses

To understand the evolutionary genetic relationship between our endolysins and other known *Bacillus* phage endolysins, we performed a phylogenic analysis of AP50-31 and LysB4 with other known *Bacillus* phage endolysins. First, sequences of 79 known *Bacillus* phage endolysins were retrieved from NCBI database. Second, we compared sequences of the retrieved *Bacillus* phage endolysins with our endolysins, and from this group of 79, we selected sequences of 23 known *Bacillus* phage endolysins to be used in subsequent multiple amino acid sequence alignments. We used two criteria for selecting which *Bacillus* phage endolysins to use in subsequent multiple sequence alignments. The first criterion was to find homologs which shared greater than 70% of the queried coverage in their amino acid sequences as listed in a BLASTP search of the NCBI protein sequence database (http://blast.ncbi.nlm.nih.gov/Blast.cgi). For the second criterion, we ensured that the searched homologs contained at least one catalytic or cell wall binding domain similar to those of endolysins AP50-31 and LysB4. Then, multiple sequence alignments with the selected homologs including our endolysins were carried out by the BLOSUM weight matrix of the ClustalW program^47^. Finally, the resultant file was subjected to phylogenic analysis using the MEGA 6.0 program (http://www.megasoftware.net/). The phylogenetic tree was constructed using the Maximum likelihood method with Jones-Taylor-Thornton model and 1,000 bootstrap replicates. Several characteristics of the putative phage endolysins and phage endolysins AP50-31 and LysB4 (molecular mass, pI, signal peptides, and transmembrane domains) were predicted by the proteomic tools available at ExPaSy (http://us.expasy.org).

### Expression and purification of recombinant AP50-31 and recombinant LysB4

*E*. *coli* BL21-pBAD-AP50-31 was grown at 37 °C in a 10 L-fermenter jar containing 5 L of Luria–Bertani (LB) medium (5 g/L yeast extract, 10 g/L tryptone, and 10 g/L NaCl) supplemented with 50 μg/mL of kanamycin with agitation at 200 rpm and aeration of 5 L/min. Recombinant AP50-31 expression was induced by the addition of arabinose (0.2%, final concentration) when the OD_600_ of the culture reached 0.5. Following 4 h of incubation at 30 °C, the cells were collected by centrifugation (6,000 × *g* for 10 min) and resuspended in lysis buffer (20 mM Tris-HCl, pH 7.5). The cells were disrupted by a conventional ultrasonic treatment for 15 min (3 s pulse with 3 s rest intervals between pulses). After debris removal by centrifugation (13,000 × *g* for 20 min at 4 °C), the supernatant was recovered and subjected to cation-exchange chromatography (SP Sepharose^®^ HP column; GE Healthcare, Little Chalfont, UK). The recovered supernatant was loaded onto an SP Sepharose^®^ HP column pre-equilibrated with buffer A (20 mM Tris-HCl, pH 7.5). The column was washed with 5 CV (column volume) of buffer A until the absorbance at 280 nm decreased to 0.05. Then, the bound AP50-31 was eluted with a linear gradient of 0 to 0.5 M NaCl in buffer B (20 mM Tris-HCl and 0.5 M NaCl, pH 7.5).

To express and purify the recombinant LysB4, the same method was used as that described above with two modifications: *E*. *coli* BL21-pBAD-B4 was used as the expression host, and the pH of the buffers used (lysis buffer, buffer A and buffer B) was 8.0.

### Development of formulations

Adequate liquid formulations to stabilise AP50-31 and LysB4 during storage or handling were developed through preformulation matrix screening. During the preformulation matrix screening, the effects on the stability of the following formulation parameters were examined (after incubation under accelerated stability study conditions (40 °C for 16 h), under vigorous constant agitation for up to 4 h, and after 12 weeks of storage at −20 °C, 4 °C, and 25 °C): pH (pH 7 *vs*. 7.5 for AP50-31 and pH 6 *vs*. 7 for LysB4); buffer type (10 mM l-histidine *vs*. 10 mM sodium phosphate); surfactant (0.5% poloxamer 188, 0.1% polysorbate 20, or 0.1% polysorbate 80); and additive (0.1% l-cysteine, 5% sorbitol, 1% sucrose, 5% mannitol, or 140 mM NaCl). All of the formulation candidates were prepared by dialyzing the elution fraction containing the phage endolysin against each formulation candidate and subsequently concentrated to 1 mg/mL using Amicon^®^ Ultra-4 10 K filters (Millipore, Billerica, MA, USA). The stability of AP50-31 and LysB4 in the formulation candidates was evaluated for their visual appearance, turbidity by absorbance at 600 nm, and three analytical assessments (SDS-PAGE, SEC-HPLC, and a biological activity assay). SEC-HPLC was performed with a BioSep™-SEC-S 2000 column (Phenomenex, Torrance, CA, USA). The mobile phase (10 mM Tris-HCl and 150 mM NaCl, pH 7.0) was applied at a flow rate of 1.0 mL/min. Next, 50 μL of each sample was injected, and the sample elution was monitored for 30 min by measuring the absorbance at 280 nm. The conventional turbidity reduction assay was used as the biological activity assay. Briefly, a 1 μg/mL dilution of phage endolysin was added to the *B*. *cereus* ATCC 4342 cell suspension (OD_600_ = 1.0). The phage endolysin dilutions were prepared using 10 mM phosphate-buffered saline (PBS; pH 7.2) solution. The time required to reach one-half of the starting absorbance (TOD_50_, equivalent to a one-half log drop in the initial concentration of viable bacteria) was determined by examining changes in the bacterial density (OD_600_) recorded every 0.5 min for 20 min.

### Expression host bacteria deposition

*E*. *coli* BL21-pBAD-AP50-31 and *E*. *coli* BL21-pBAD-B4 were deposited in the Korean Collection for Type Cultures (KCTC; Jeongeup, Republic of Korea). The deposition numbers for *E*. *coli* BL21-pBAD-AP50-31 and *E*. *coli* BL21-pBAD-B4 are KCTC 12708BP and KCTC 12709BP, respectively.

### *In vitro* bacteriolytic activity test against *Bacillus* strains

The *in vitro* bacteriolytic activity test was performed against strains belonging to the *Bacillus* genus by a conventional turbidity reduction assay under the same conditions described above except for the final phage endolysin concentration (5 μg/mL). When the TOD_50_ could not be determined within 20 min, the endolysins were added in large amounts at a final concentration of 300 μg/mL. If the TOD_50_ could be determined at a final concentration of 300 μg/mL, this strain was marked as ND (not determined) because it is susceptible, but the exact TOD_50_ value could not be determined (≤300 μg/mL; >5 μg/mL). If not, it was marked as NS (not susceptible). The non-*anthracis Bacillus* bacterial strains tested are shown in Table 1.

### *In vitro* bacteriolytic activity test against *B*. *anthracis*

*In vitro* bacteriolytic activity tests were performed against four *B*. *anthracis* strains (*B*. *anthracis* ΔSterne, *B*. *anthracis* Sterne, *B*. *anthracis* ATCC 14578, and *B*. *anthracis* HYU01). Strains of *B*. *anthracis* were grown on LB agar plates at 35 °C overnight. The next day, a single colony from each plate was inoculated into 20 mL brain heart infusion (BHI) medium in a 50-mL CELLSTAR^®^ CELLreactor™ tube (Greiner Bio-One, Austria) and incubated at 35 °C with shaking (200 rpm) for 6 h. Then, 200 μL of the culture broth was transferred to 20 mL BHI medium in a 50-mL CELLSTAR^®^ CELLreactor™ tube for overnight incubation at 35 °C with shaking (200 rpm). The next day, 100~200 μL of the culture broth was transferred to 20 mL BHI medium in a 50-mL CELLSTAR^®^ CELLreactor™ tube until the culture reached the exponential growth phase. Then, the culture broth was harvested by centrifugation at 3,000 × *g* and 4 °C. The cell pellet was washed three times with ice-cold PBS solution. Then, the cell pellet was resuspended in ice-cold PBS solution to an OD_600_ of 1.0. The endolysins were added at a final concentration of 5 μg/mL, and the change of OD_600_ was monitored at 30 s intervals for 20 min to determine the TOD_50_. All tests using *B*. *anthracis* were approved by the institutional biosafety committee and conducted in a biosafety level 3 laboratory following the regulations of the Republic of Korea.

### *In vivo* efficacy test of endolysin LysB4 against *B*. *anthracis*

Six-week-old female A/J mice were purchased from Japan SLC Inc. (Shizuoka, Japan) and used in this experiment after 7 days of acclimation. All of the mice were maintained in the experimental facility with unrestricted access to a rodent diet and water. All animal care and experiments were approved by the Institutional Animal Care and Use Committee of the Agency for Defense Development and performed according to the relevant regulations and guidelines issued by the Republic of Korea. The intranasal infection model was used to test the *in vivo* efficacy of endolysin LysB4 against *B*. *anthracis*. Briefly, the mice were anaesthetised by the intraperitoneal injection of 90 mg/kg ketamine (Yuhan Co., Seoul, Republic of Korea) and 10 mg/kg xylazine (Bayer Korea, Seoul, Republic of Korea), and 25 μL of spore suspension (1 × 10^7^ CFU/mL) was inoculated intranasally (G1-G5). The inoculated dose was approximately four times the 50% lethal dose that had been reported previously^48^. To check the survival rate, clinical signs, and body temperature change, the mice were randomly assigned to three groups (G1–G3, *n* = 8). At 6, 24, and 48 h post-infection, the animals were anaesthetized, and 25 μL buffer (G1) or endolysin LysB4 (G2: low dose group, 10 μg/head; G3: high dose group, 100 μg/head) were instilled intranasally. The survival rate and clinical signs of each experimental group were monitored every 24 h up to 14 days post-infection, and the body temperature was measured every 24 h up to 7 days. To compare the bacterial count in tissues between the buffer- and endolysin-treated groups, the animals were randomly allocated and intranasally treated with buffer (G4) or endolysin LysB4 (G5, 100 μg/head) at 6, 24, and 48 h post-infection (8 mice per group). Three days after the bacterial challenge, the mice were euthanized, and the lungs, livers, spleens, and kidneys were collected in sterile PBS solution to count the bacteria. Each tissue sample was weighed, homogenised, and serially diluted, and 100 μL of each homogenate dilution was plated onto tryptic soy agar plates. The number of bacteria (CFU/g tissue) was calculated followed by the overnight culture of plates at 37 °C. For histopathological observations, parts of the lungs, livers, spleens, and kidneys were placed in 10% neutral buffered formalin, processed, and embedded in paraffin. Then, 3-μm-thick sections were prepared. After haematoxylin and eosin staining, the prepared tissue sections were examined under light microscopy. Tissues from non-infected and non-treated control mice were also prepared to compare the histopathological findings.

### Statistical analysis

All statistical calculations were performed using Prism (GraphPad, La Jolla, CA, USA). The results of survival rates and tissue bacterial loads were analysed by the Mantel–Cox test and 2-way analysis of variance, respectively (*P < 0.05; **P < 0.01; ***P < 0.001).

### Data availability

The data that supports the findings of this study are available in the article and the supplementary information or from the corresponding authors on request.

## Electronic supplementary material

Supplementary information
