# Supplementary material for: Characterisation of the antibacterial properties of the recombinant phage endolysins AP50-31 and LysB4 as potent bactericidal agents against Bacillus anthracis
Source: Sci Rep. 2018 Jan 8;8:18. doi: 10.1038/s41598-017-18535-z (PMC5758571; doi:10.1038/s41598-017-18535-z)
Supplement: Supplementary file 1 — Supplementary information [file 41598_2017_18535_MOESM1_ESM.pdf]

Characterisation of the antibacterial properties of the recombinant phage endolysins AP50-31 and LysB4 as potent bactericidal agents against *Bacillus anthracis*

Sangjin Park[1,2 #], Soo Youn Jun[3#], Chang-Hwan Kim[2], Gi Mo Jung[3], Jee Soo Son[3], Seong Tae Jeong[2], Seong Jun Yoon[3], Sang Yup Lee[1*] & Sang Hyeon Kang[3*]

[1] Metabolic and Biomolecular Engineering National Research Laboratory, Department of Chemical and Biomolecular Engineering (BK21 Plus Program), Center for Systems and Synthetic Biotechnology, Institute for the BioCentury, Korea Advanced Institute of Science and Technology (KAIST), Daejeon 34141, Republic of Korea. [2] The 5th R&D institute, Agency for Defense Development (ADD), Yuseong P.O.Box 35-5, Daejeon, 34186, Republic of Korea. [3] iNtRON Biotechnology, Inc., Room 903, JungAng Induspia V, 137, Sagimakgol-ro, Jungwon-gu, Seongnam-si, Gyeonggi-do 13202, Republic of Korea. Correspondence and requests for materials should be addressed to S.H.K. (email: kangsh0403@naver.com) or S.Y.L. (email: leesy@kaist.ac.kr)

# Sangjin Park and Soo Youn Jun equally contributed in this study.

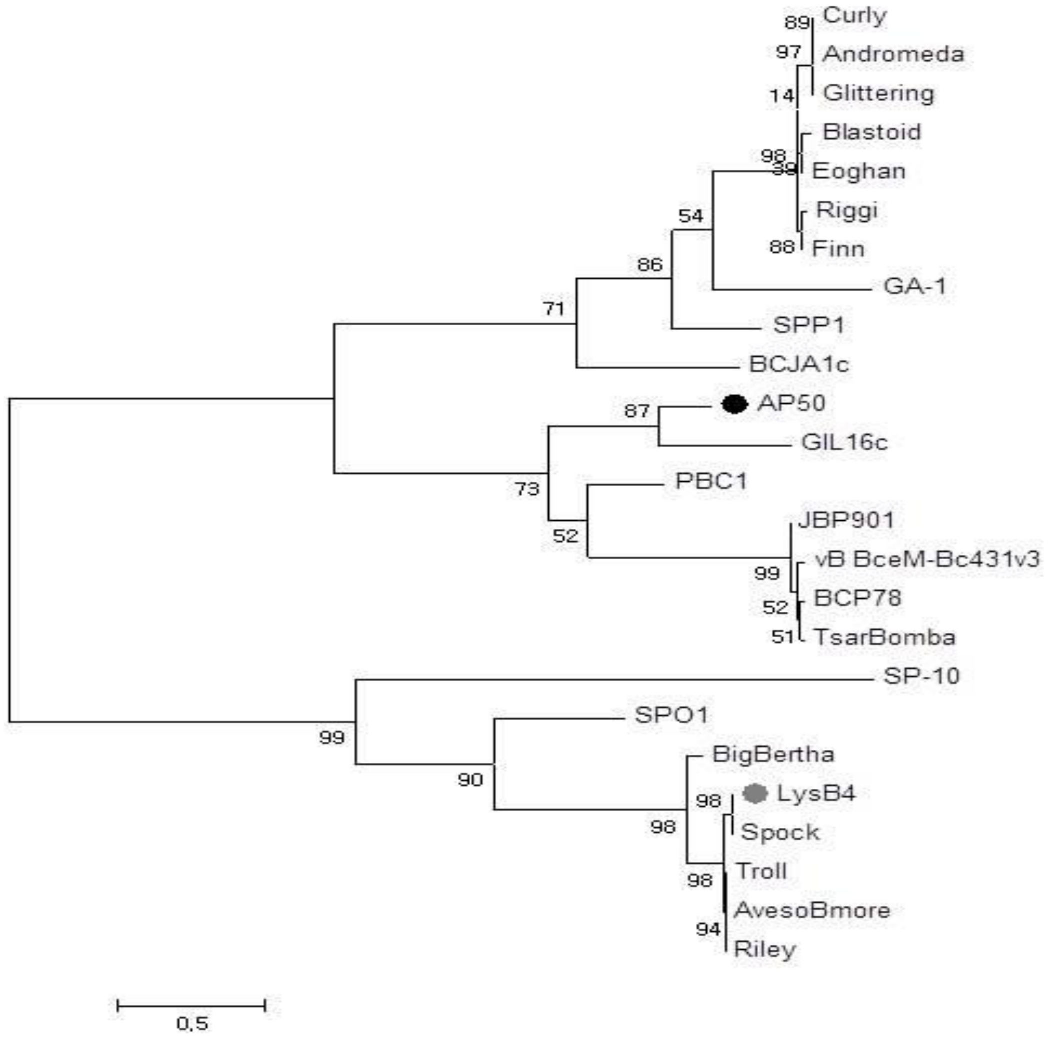

Figure S1. Phylogenic analysis of the phage endolysins. The phylogenetic tree was constructed using a Newick file and displayed using the MEGA 6.0 program. The black-filled and grey-filled circles indicate phage endolysins AP50-13 and LysB4, respectively. Bootstrap values (indicated on nodes) are shown for selected internal branches. The bar is a marker of branch distance length.

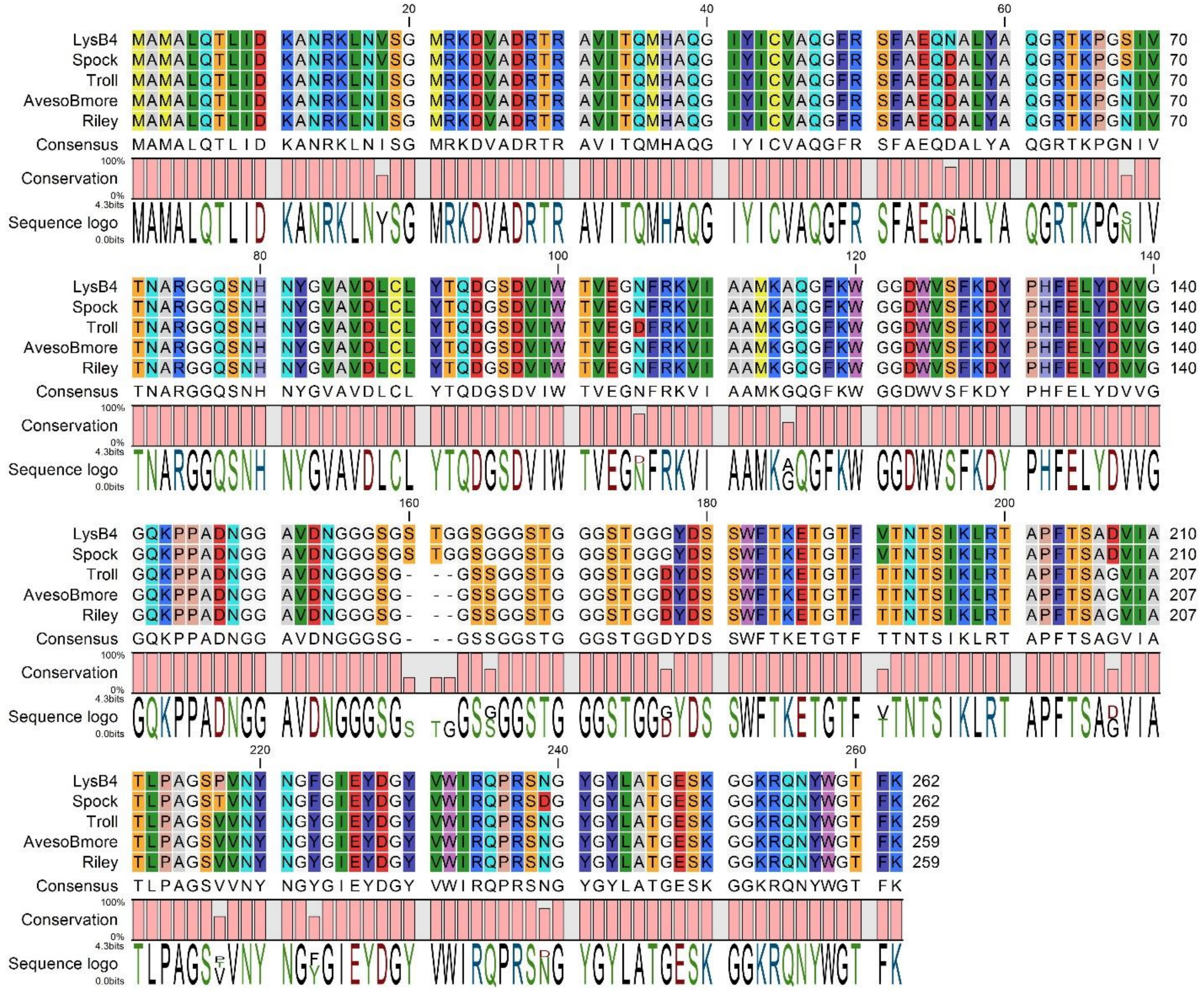

Figure S2. Alignment of amino acid sequences of endolysins LysB4, Spock, Troll, AvesoBmore, and Riley. The amino acid sequences of endolysins AvesoBmore and Riley were determined to be 100% identical to each other, while those of endolysins LysB4 and Spock differed by one amino acid. The alignment was performed using CLC Main workbench (version 7.7.3; CLC bio, Aarhus, Denmark).

Table S1. Characteristics of the putative endolysins of phages infecting *Bacillus* species.

Host	Phage name	Protein ID	Size (amino acid)	MW (kDa)	pI	Conserved domain (s)			Phage classification
						N-terminal	Middle	C-terminal	
B. anthracis	AP50	YP_002302543.1	252	27.7	6.44	Amidase_3		Amidase02_C	*Tectiviridae*
B. cereus	B4	YP_006908235.1	262	27.9	9.21	VanY		SH3_5	*Myoviridae*
B. cereus	BCP78	AEW47021.1	272	29.2	6.65	Amidase_3		SH3_5	*Myoviridae*
B. cereus	JBP901	YP_009149049.1	272	29.3	7.04	Amidase_3		SH3_5	*Myoviridae*
B. cereus	vB_BceM-Bc431v3	YP_007676909.1	272	29.4	6.38	Amidase_3		SH3_5	*Myoviridae*
B. cereus	PBC1	YP_006383478.1	254	27.2	5.94	Amidase_3		Amidase02_C	*Siphoviridae*
B. thuringiensis	AvesoBmore	YP_009206412.1	259	27.7	9.07	VanY / Peptidase_M15_4		SH3_5	*Myoviridae*
B. clarkii	BCJA1c	YP_164437.1	355	39.1	9.74	Amidase_3		PG_binding_1	*Siphoviridae*

B. pumilus	Blastoid	YP_008771857.1	299	32.1	9.81	Amidase_3	LysM	LysM	*Siphoviridae*
B. pumilus	Riggi	YP_008770590.1	303	32.6	9.81	Amidase_3	LysM	LysM	*Siphoviridae*
B. pumilus	Glittering	YP_008770668.1	299	32.2	9.87	Amidase_3	LysM	LysM	*Siphoviridae*
B. pumilus	Finn	YP_007517651.1	303	32.6	9.89	Amidase_3	LysM	LysM	*Siphoviridae*
B. pumilus	Eoghan	YP_007517421.1	299	32.1	9.82	Amidase_3	LysM	LysM	*Siphoviridae*
B. pumilus	Curly	YP_007517576.1	299	32.2	9.92	Amidase_3	LysM	LysM	*Siphoviridae*
B. pumilus	Andromeda	YP_007517497.1	299	32.3	9.89	Amidase_3	LysM	LysM	*Siphoviridae*
B. subtilis	SPP1	NP_690702.1	271	29.9	9.67	Amidase_3			*Siphoviridae*
B. subtilis	SPO1	YP_002300379.1	343	37.1	9.78	VanY	PG_binding_1	PG_binding_1	*Myoviridae*
B. subtilis	SP-10	YP_007003388.1	236	25.9	9.81	Peptidase_M15_4		PG_binding_1	*Myoviridae*
B. thuringiensis	GIL16c	YP_224129.1	288	32.1	7.15	Amidase_3		SPOR	*Tectiviridae*
B. thuringiensis	TsarBomba	YP_009206875.1	272	29.2	6.37	Amidase_3		SH3_5	*Myoviridae*
B. thuringiensis	Spock	YP_008770279.1	262	27.9	8.92	VanY		SH3_5	*Myoviridae*
B. thuringiensis	Riley	YP_009055819.1	259	27.7	9.07	VanY /		SH3_5	*Myoviridae*

						Peptidase_M 15_4			
B. thuringiensis	BigBertha	YP_008771084.1	312	33.7	8.68	VanY / Peptidase_M 15_4	SH3b	SH3b	*Myoviridae*
B. thuringiensis	Troll	YP_008430843.1	259	27.7	8.91	VanY / Peptidase_M 15_4		SH3_5	*Myoviridae*
Bacillus sp.	GA-1	NP_073698.1	239	26.4	9.70	Amidase_3		LysM	*Podoviridae*
